# Supplementary material for: Content-rich biological network constructed by mining PubMed abstracts
Source: BMC Bioinformatics. 2004 Oct 8;5:147. doi: 10.1186/1471-2105-5-147 (PMC528731; doi:10.1186/1471-2105-5-147)
Supplement: Additional File 2 — The original results of the above study (non-essential files are deleted to keep the file size under the limit set by BMC bioinformatics). [file 1471-2105-5-147-S2.bz2 › chilibotAdditionalFile2/dip40/156ID8438581E558/html/N_M2.html]

 


 **N** and **M2** 
  
Found 5408 abstracts in PubMed, retrieved 40.  
 

 What does Google say? 
 PDF only 
| .edu only 

---

**Interactive relationship** (e.g. stimulation, inhibition, etc)

**Neutral relationship**- Allosteric site on muscarinic acetylcholine receptors identification of two amino acids in the muscarinic  **m2**  receptor that account entirely for the  **m2**  m5 subtype selectivities of some structurally diverse allosteric ligands in  **N**  methylscopolamine occupied receptors.  Ref: 12815157 Mol Pharmacol, 2003
- Two epitopes have been identified recently to be responsible for the high affinity binding of alkane bisammonium and caracurine V type allosteric ligands to  **N**  methylscopolamine NMS occupied  **M2**  muscarinic acetylcholine receptors, relative to M5 receptors the amino acid  **M2**  Thr423 at the top of transmembrane region TM 7 and an epitope comprising the second extracellular loop o2 of the  **M2**  receptor including the flanking regions of TM4 and TM5.  Ref: 12815157 Mol Pharmacol, 2003

**Non-interactive relationship** (e.g. studied together, co-existance, homology, etc.)

- Although cysteine substitution mutations to residues in the  **N**  terminal half of the alpha subunit  **M2**  M3 loop dramatically impaired the gating efficacy, the same mutations exerted little effect when incorporated into corresponding positions of the beta subunit.  Ref: 12871591 J Neurochem, 2003
- The filters were  **n** =10 PF1000N effective membrane surface 0.15  **m2** .  Ref: 12823420 Artif Organs, 2003
- 8 CdSO4 topology two new supramolecular isomers of  **M2**  bdc 2 L 2  **n**  coordination polymers.  Ref: 12841232 Chem Commun Camb , 2003
- In the narrow range of decreased mean ADC values 0.60 to 0.40 x 10 9  **m2**  x s 1, a 33% decrease in mean ADC is associated with a 122% increase in lactate  **N**  acetyl aspartate ratio.  Ref: 12817104 Stroke, 2003
- The unique six amino acid  **N**  terminal extension of Gqalpha when added to the  **N**  terminus of Gi1alpha neither prevented  **M2**  receptor coupling nor permitted M1 receptor coupling.  Ref: 12809511 Biochemistry, 2003
- DESIGN To this goal, in a large population  **n**  = 23 39 men, 98 women, age range 20 80 years, body mass index BMI range 26.4 4.3 kg  **m2**  of well nourished adults with total anterior pituitary deficit including severe GHD as shown by a GH peak below the 1st centile limit of normal response to GHRH ARG tests and or ITT we evaluated the diagnostic value of a single total IGF I measurement.  Ref: 12807504 Clin Endocrinol Oxf , 2003
- Mean glomerular filtration rate decreased from 58 19 at 1 year  **n** =42 to 44 16 mL min 1.73  **m2**  body surface area at 10  **n** =33 years.  Ref: 12883184 Transplantation, 2003
- In addition, treatment with antioxidant  **N**  acetyl L cysteine inhibited anti Id induced apoptosis in G1 2 and  **M2**  10.  Ref: 12846046 Clin Exp Rheumatol< MedlineTA>Clin Exp Rheumatol, 2003
